# Hidden from history: Carriage cleaners in the United Kingdom from 1849 to COVID-19

**DOI:** 10.1177/00225266231156114

**Published:** 2023-02-13

**Authors:** Julia Winterson

**Affiliations:** 8748 University of York, UK

**Keywords:** Carriage cleaners, railway workers, female employees, nationalisation, privatisation

## Abstract

The story of carriage cleaners has been sadly neglected in the history of railway workers. The work has low pay, it is sometimes unpleasant, and it is also physically tough. This panorama paper explores some of the literature surrounding the history of carriage cleaners from the earliest records in the nineteenth century, through the two world wars, up to the early days of privatisation. The focus is on female carriage cleaners, exploring the reasons why their work has been hidden from history and putting forward an argument as to why more attention should be drawn to it. Despite its low pay and poor working conditions, cleaning work is found at an important nexus of the railway economy, ensuring that the spaces of railway travel remain sanitary and functional. Given their importance for the operation of the railway transport system, it is surprising that cleaners remain largely invisible.

## Dirty work, hidden from sight

Carriage cleaning, traditionally undertaken by some of the poorest people in the UK, is riven with political issues affecting, for example, their work conditions, wages, and hours. As such it relies on the intersecting inequalities and oppression of class, gender, and race. Although little has been written about carriage cleaners, in contrast, a good deal has been written about the work of engine cleaners – a man's job and traditionally the entry step on the career path to being an engine driver. There was nothing radically different about the two jobs; both were heavy, dirty, and sometimes dangerous work, and both were “men's jobs” until the two world wars when women took on these roles. From the twentieth century, there were significant numbers of women within the body of cleaners. During the 1980s and 1990s, women were underrepresented in many areas of the railway sector, but preponderant in cleaning work. Through a synopsis of previous research, the paper explores the support of the National Union of Railwaymen (NUR) in campaigning for better work conditions for cleaners and equal pay for women and finds them wanting. Privatisation brought about profound changes through its fragmentation of the industry and the dismantling of British Rail (BR). The consequent outsourcing had negative effects on pay and work conditions for cleaners.

The work of carriage cleaners is largely hidden from sight. Leigh-Star argues that there are many aspects to the study of infrastructure and that some of these, because of their potential “invisibility”, have often been ignored.^1^ As she writes, “with any form of work, there are always people whose work goes unnoticed or is not formally recognised” citing cleaners, amongst others, by way of example. She warns that leaving such workers out can lead to a “nonworking system”; studying the unstudied, she argues will “stretch our understanding of identity, status and community” and underpin a “social justice agenda by valorizing previously neglected people and things”.^2^ In their article “Out of Order” Graham and Thrift argue that the processes of maintenance and repair are what keep modern societies going, but they have been neglected by most commentators as “somehow beneath their notice”; instead they should be given the systematic attention that they deserve and should not be written off as “simply mundane or slavishly repetitive” incidental activities.^3^ Over 150 years earlier, Sir Francis Head made a similar point in the preface to Stokers *and Pokers*, which included his observations on the London and North Western Railway.^4^ As he wrote, “When the railways were first established, every living being gazed at a passing train with astonishment and fear” but that the “nine days wonder” soon passed and trains were taken for granted.^5^

In recent years, there has been an upsurge of research into what is referred to as “dirty work” that which stigmatizes work and workers. Rabeloa and Mahalingamb list three types of “dirty work”, physical, social, and moral. Cleaning belongs to the physical category where the “work involves trash, bodily fluids, death, and/or dangerous conditions”. They argue that when literal dirt is involved, the dirt “stains” those who are connected with it, stigmatizing the workers when the negative qualities associated with dirt are projected onto them. Because “many societies abide by cultural norms that equate cleanliness with goodness” this can result in avoidance of people, and objects that are perceived as “dirty”. They argue that creating distance from the pollution of dirt and from those who deal with it can render “dirty” occupations as invisible. Paradoxically, occupations in “dirty” work are often regarded as necessary for “societal effectiveness” at the same time as being viewed as demeaning, disgusting, and/or undesirable forms of work.^6^

As a result, of this avoidance, “dirty” occupations dealing with physical dirt are often carried out by those who are separated socially from other groups. As Simpson and Simpson write this means that “Dirt and dirty work are therefore tied up with a moral and social order with implications for prestige, work hierarchies, and social positioning”.^7^ Carriage cleaning was undertaken by the working classes from the twentieth century onwards, predominantly by women and migrant workers^8^ whose invisibility was therefore a social construction, reflected and reinforced by boundaries across class, gender, and ethnicity.

The main focus of this paper is on female carriage cleaners. The socialist historian Sheila Rowbotham was one of the first to write at length about the ways in which the history of women is often hidden from view. In *Hidden from History* (1973), a documentation of women's history from the seventeenth century onwards, she argues that what little is written details women's oppression through sexism and class, and their fight for equality.^9^ In terms of women's railway work specifically, very little has been written, reinforcing Margaret Walsh's statement that transport history was written by men for a male audience.^10^ It is also difficult to find data on the roles and distribution of men and women in the railway workforce or, as Anne Munro observes, the position of women in unions.^11^ Caroline Criado-Perez argues that this gender data gap is universal; it creates a gap in our knowledge and is at the root of systematic discrimination against women.^12^ In the absence of relevant information in railway histories, Helena Wojtczak's *Railwaywomen: Exploitation, Betrayal and Triumph* makes much use of primary sources*,* tracing the development of women's employment and charting the resistance women faced in a predominantly male industry. Because the work of cleaners has such low-status women were not well-represented in the traditional power structures such as management and trade union officialdom.^13^ Wojtczak documents some of the negative attitudes that railway management had towards women employees and gives an outline of women's membership and representation in the railway unions. In contrast, Philip Bagwell's critical analysis of the work of the NUR falls short when detailing the union's attitudes and policies towards both women and cleaners.^14^

In conclusion, the work of carriage cleaners is of low status and therefore hidden from sight. This invisibility is a social construction where class, gender, and race, further compounded by the “dirtiness” of the work, lead to systematic discrimination. Partly as a consequence, little has been written on the topic. An argument has been made for further research into infrastructures, carriage cleaning would fall into this category. There is also scope for further research into female carriage cleaners specifically where little data is currently available.

## Carriage cleaning in the nineteenth century

A rare account of the work of carriage cleaners appeared in 1849 when Sir Francis Head published a small volume, *Stokers and Pokers*. As he wrote, “On the practical working of the railway there is no book extant, nor any means open to the public of obtaining correct information on the subject”.^15^ Head's observations on carriage cleaners are based on a visit to Euston station that year. He noted that on arrival at the station, trains are removed to the sidings for the work to take place.

A large gang of strong he-housemaids, clattering towards them in wooden shoes and in leather leggings rising above their bony knees, are seen advancing; some with mops in their hands, others with large chamois leathers, while others are carrying on their shoulders a yoke, from which are suspended *in equilibrio* two pails … water is immediately drawn off, and the busy operation of washing then begins. Half a dozen dusty … dirty-bodied carriages are simultaneously assailed on each of their sides by wet mops flying up, down, and around in all directions.

Once the carriages have been washed and dried, they are examined by a foreman and then they are visited by “the duster” who goes inside each carriage to clean the windows, wipe the woodwork, and brush the seats. Head's report took place at a time when carriages were segregated into first-, second-, and third-class carriages. The cleaners had a similar delineation. According to Head, “A ‘First-class mopper’ would on no account demean himself by mopping a second-class carriage”. Similarly, a “Second-class mopper” only attained that distinction after he had served sufficient time mopping “horse boxes and common luggage trains”.^16^ Third-class carriages were cheap but unpleasant to travel in; in the 1840s, they were little more than overcrowded low-sided wagons. A measure of this unpleasantness is that in 1873, South Eastern Trains installed iron funnels into the third-class carriage floors to make it easier for the cleaners to dispose of spittle.^17^

Head's account is an unusual piece of railway history in that it includes a description of the work of carriage cleaners. In contrast, a good deal has been written about the work of engine cleaners – traditionally the entry step on the career path to being an engine driver. Engine drivers were considered to be the elite of the railway workforce, whereas carriage cleaners were seen as the lowest of the low. This is well illustrated in the index to McKenna's 1976 exploration of railway workers where the entry for the word “cleaners” does not mention carriage cleaners but redirects the reader to “engine cleaners” where there are 14 entries.^18^ In her study of the infrastructures of information systems, Leigh-Star writes of them employing “what literary theorists would call a master narrative or a single voice that does not problematize diversity”. The same could be said here where the master narrative focuses on the work of engine cleaners and side-lines the work of carriage cleaners. In doing so this risks overlooking potentially valuable insights into what Leigh-Star refers to as “workplaces, materiality and interaction”. The inclusion of carriage cleaners has the potential to, in Leigh-Star's words, “valorise previously neglected people” and lead to a better understanding of “identity, status and community”.^19^ Like most Victorian employers, railway companies gave the jobs with the least prospects and the lowest status to women, so engine cleaning, with its prospect of becoming an engine driver, was not available to them. During the Victorian and Edwardian periods, neither was carriage cleaning.

This was a period when there were many constraints on the work of women in terms of femininity and the domestic ideology, the patriarchal household and the breadwinner norm. As Jane Humphries puts it, gender was “defined in terms of the role of housewife and mother and its mirror image, the breadwinner male”.^20^ Ellen Jordan argues that domestic ideology laid down stringent conditions which needed to be met for work to be considered “feminine” and that these conditions are evident from the “reactions of middle-class philanthropists to the work undertaken by working-class women”. The work should not involve contact with “industrial grime … or baring the body or the wearing of trousers”. Furthermore, it should not involve “continuous muscular effort” nor should it be undertaken in the company of men. Jordan goes on to write that such criteria lay behind some Government legislation citing, amongst other examples, the legislation forbidding the employment of women in underground mines. This, she argues, was the motivation rather than a consideration for women's health.^21^

Nevertheless, there were a small number of female carriage cleaners in the nineteenth century, although their cleaning work was restricted. This was partly because the work took place in sidings amongst moving engines and wagons which was looked upon as too dangerous an environment for what the Victorians saw as the “fairer sex”. Furthermore, the women were restricted by the nature of their clothes; their long skirts and tight corsets made climbing into carriages difficult, not to say dangerous and rendered it almost impossible for them to climb onto carriage roofs to fill lavatory water tanks and tend to carriage lamps.^22^

Because the work of carriage cleaners has so often been side-lined, in contrast with that of engine cleaners, Heads's account provides a rare and illuminating piece of social history. Further research into the work of nineteenth-century women in this field has the potential to lead to a better understanding of women's identity, status, and community.

## Accidents, hazards and two world wars

In 1913, there were 214 female carriage cleaners who were paid 15 shillings a week, much less than their male counterparts who were able to work in the sidings and climb on top of the carriages. The men were paid 21 shillings a week – 42 per cent more.^23^ That year 1,500 railwaymen marched through the streets of Willesden to Gladstone Park to protest against the employment of underpaid women by the Great Western Railway Company. One of the speakers, Mr Church said that by employing women carriage cleaners at 15 shillings a week the Great Western Railway (GWR) were “not only contravening the Conciliation Bill but were causing women to displace men whose minimum weekly wage was one guinea. The age of chivalry is not dead among the working classes’, ‘and we are determined not to see the labour of our women folk exploited by a wealthy company’”. Another speaker, Councillor Dobb, said that “to employ women at carriage cleaning was to degrade the women”.^24^ These are perhaps mixed messages, but both take a paternalistic approach which seeks to reinforce the hierarchical gender system under the guise of the protection of “our” womenfolk.

Until World War I nearly all of the carriage cleaners were men, but with so many men away fighting there was a greatly increased demand for labour. This meant that women were called upon to fulfil some of the roles in the railway, and carriage cleaning was one of the first jobs where women were substituted for men.^25^ By 1916, there were over 2,000 female carriage cleaners. The restrictive clothing problem was eventually overcome: corsets were loosened; skirts were shortened; and then very daringly for the time, trousers were worn. Once they received official approval, trousers were issued to the women by railway companies.^26^

Carriage cleaning continued to be undertaken largely by hand using buckets and long-handled brushes. It usually took place at ground level but sometimes from staging. Routine cleaning involved the same methods that Head had observed in 1849: washing down the exteriors; sweeping the floors; and dusting the interior woodwork. Once a month, the trains were given a more thorough cleaning. To remove the grime and grease, the exteriors were treated with an acid and glycerine solution, which was applied by brush, rubbed in and washed off with water. The use of acid clearly posed health risks to the cleaners, but these were not the only risks they encountered. Working on the line, often amongst moving trains, was also very dangerous. Cleaners were required to cross the lines, to work between the carriages and on top of them. There are numerous accounts of accidents, sometimes fatal, which befell carriage cleaners throughout the nineteenth century and well into the twentieth century. One of the earliest accounts of the death of a carriage cleaner at work comes from 1855 when Charles Rollings was accidentally squeezed between the buffers of two carriages at Worcester Station.^27^ This was not a singular event; John Fox suffered the same fate at Brighton Railway station in 1905 when he was “caught between the buffers of a stationary carriage and another which was sent forward by an engine shunting other carriages in front of it”.^28^ Moving trains were hazardous; in 1844, a 24-year-old GWR carriage cleaner named Westmitt was knocked down and killed at Addison Road Station in Kensington. Some cleaners received compensation for injuries at work but this was not guaranteed. In 1903, William Howden “had both legs cut off at Princes Street Station … while he was engaged picking pieces of paper off the line”. He sued the Caledonian Railway Company for damages and was awarded £78 under the Employers’ Liability Act. The railway company appealed but they lost.^29^ Wartime put further dangers in the way of railway workers with, for example, carriage cleaners having to work in dimly lit yards.

The inter-war years saw a shift back to the pre-war position with the number of female carriage cleaners being reduced and replaced by men who had returned from the war. By 1923 there were only 882 women but 6,829 men doing the job.^30^ Matheson writes that the railway institutions struggled to come to terms with female labour on the railways, feeling a need to protect the interests of the railway men citing *The Railway Gazette* with its patriarchal perspective

That the employment of women on the railways of this country has contributed in no small degree to the maintenance of and efficient transport service cannot be gainsaid … they have shown a natural aptitude for carriage cleaning … [this] is the work in the satisfactory fulfillment of which women most nearly approach men, but even here it is questionable whether the work formerly performed by a given number of men can be covered by the same number of women; six females to five males has been reckoned a fair comparison.^31^

This perceived inferiority in the work of the women was reflected in their pay. In 1918, the “average earnings” of a male carriage cleaner were 60s 11d and for women 40s 1d (roughly 66 per cent of the men's pay).^32^ Women continued to be paid at a lower rate and the NUR did not uphold their claims for equal pay.

At the start of World War II, the precedent of women replacing absent men had already been set and by 1942, there were 3,426 female cleaners. Wojtczak includes an account of the work of Victoria Simpson, a carriage cleaner at Victoria Station in London during the war. Her job included cleaning inside and outside the trains, cleaning the signal lamps and tanking. Tanking meant “climbing on to the roof of the trains with a hose pipe and filling the water tanks for the toilets and basins … through a hole in the roof”. It was clearly a dangerous procedure.

We used acid to clean the outside of the trains using a brush on a long stick … the acid stayed on for about an hour by which time we had covered an eight coach train. Then we had to wash it off, sometimes in the winter the acid and water froze on the windows and it was hard work, we couldn’t use hot water, the windows would have broke. The only hot water we had was an old copper that we kept going with coal from the engine drivers, and that was for scrubbing floors and sides. One lady went through with raw soap and … a second would come along rinsing, bending down all the time right through the train, and a third to dry it up … We only earned about £4 and we had to do shift work in between dodging the Doodlebugs … We often had to walk over the live lines. All in all we worked very hard.^33^

The National Archive of Railway Oral History housed at the National Railway Museum includes several interviews with women who worked on the railway during World War II. These interviews are central to Susan Major's 2018 book *Female Railway Workers in World War II*.^34^ Florence Brinklow joined LNER in 1940; her friend told her that they were taking on women to clean the trains at the BR car sheds in Ilford. “I said, I’ll have a go. Anyway, I went down there and I got the job and I started the same night because I think they were short of women”. As well as taking place during anti-social hours, the work was fraught with danger: she remembers working there during air raids and was there when King's Cross station was bombed. She also had several accidents but did not claim compensation for her time off work for fear of losing her job.^35^

We all worked in different little gangs. There were about seven in a gang but that was to clean the whole train. I had several accidents in Ilford. In one accident I got a fractured pelvis and a broken arm when I was climbing up a ladder on to the carriage roof. It is all different now, the carriages are raised. I was on night work and it was a very cold February night and I was climbing up the ladder and when I got to the top of it I slipped. It came down and I got a fractured pelvis and a broken arm. I had an awful lot of accidents; there were stop cocks for the water supply and they used to leave the top off so, of course if you are not aware of it, over you go, and that happened to me quite a few times. I got a whiplash once. When I got the fractured pelvis and a broken arm I was off for quite a while. They said to me why don’t you claim compensation, but I didn’t. I was afraid that I might lose my job. When I was able to I had to go to Shenfield and see the governor and he asked if I would like a job on the platform, so I said I would give it a try.^36^

Laura Scott was born in 1911. She lived in York, working in 1941 as a carriage cleaner. The work was all done by hand and there was no protective clothing other than gloves.

We were cleaning the car seats, the ones which had never been cleaned properly before. We had a bucket with special stuff in it, you rubbed it on and then you had to rub it with a scourer until it became clean. I enjoyed it. It took a long time; you would only manage about half a carriage in a morning. You had to rub it until it was dry. The few that we did must have stood out because they were so clean. I think of these ladies in London, all dressed up going to work, sitting on filthy seats. It wasn’t dirty where we worked but it was dusty. We had some gloves on but you had to wear your own clothes. I worked cleaning carriages for about two years but then I got married again so I had to leave.^37^

Scott refers to the “ladies in London, all dressed up going to work” sitting on dirty seats. Explicit references to cleanliness were rarely made in railway advertisements, but it could be argued that cleanliness was an implicit feature with pictures of smartly dressed people sitting in clean carriages emphasised by the use of bright vivid colours. As Colin Divall writes in his study of the marketing of Britain's passenger trains, because the train “metaphorically sold the idea of railing … right from their foundation in 1923, the Big Four emphasised the train's capacity for civilised mobility as in this LMR advertisement: ‘Punctual train services, clean and healthy carriages, good meals nicely served, civility and attention, will be known as the distinguishing characteristics of the London, Midland and Scottish Railway’”.^38^ Divall sees this as promoting the train “as a bastion of bourgeois respectability” an idea which echoes Scott's perception.^39^

Scott mentions having to leave when she got married; this was standard practice for many workers across different professions at the time, hence her note of acceptance. In the words of another interviewee, Maureen Evans, a clerk at London, Midland & Scottish Railway in Crewe, “In those days of course it was traditional that if a female married that they had to leave the railway” in the words of Doreen Dickinson, a goods clerk in Liverpool, “They just notified you that when you got married you had to leave”.^40^ Several arguments were made for the marriage bar, all, it could be argued, rooted in Victorian middle-class moralism with its patriarchal ideal of womanhood – one of wife and mother, the pivot of the family and the guardian of Christian virtues. The idea persisted that the most important role of a woman was to look after her husband and bear children so it would not be possible for her to successfully fulfil a paid work position at the same time. This idea of reproductive labour inevitably resulted in the gendered division of labour and the perpetuation of women's subordination where they were restricted to jobs with lower pay and status.

A further reason for the marriage bar was to keep the jobs for the men. This was enshrined in law by the Restoration of Pre-War Practices Act 1942 stating that after the war women would have to give up “‘men's jobs’ to accommodate returning servicemen”.^41^ It was clearly true in the case of Hilda Glassey who worked as an engine cleaner, traditionally a man's job, during World War II, a job which she enjoyed, only to have to give it up at the end of the war. Hilda Glassey was born in 1908 in Bournemouth. When she left school she worked in a shop, next a hotel, and then as a school dinner lady, but during World War II, in 1941, she got a letter from the Labour Exchange giving her a job as an engine cleaner.

It was a big change and a very dirty job but I was very happy. We went to the central station in Bournemouth [Bournemouth Central] where we had to clean the engines and the flues with a long wire brush. It was very dirty but we enjoyed the work very much. We had eight girls and worked every day, except Sundays, from 8.00 until 4.00. I was there until the war ended and all the men came back so we weren’t needed. I was in charge of all the women, they did what they were told, there were no problems. We had overalls, they were provided, and hats. It was a shock getting on top of the engines, I got stuck once, I enjoyed the work, it was quite an experience.^42^

Post World War II, when men returned from the military field, the number of women employed by mainline railways reduced from 95,061 (1945) to 58,085 (1946). Furthermore, although the work of carriage cleaning was now deemed acceptable for women, the same was not true of engine cleaning.^43^

The interviews with women of this generation, one that has almost died out, offer glimpses into the dirty and dangerous nature of the work conditions. The unfair treatment of women who worked as carriage cleaners during this period represents a microcosm in the macrocosm of wartime roles for women; it begs many questions where further research and critical analysis could lead to valuable insights into, for example, attitudes towards unequal pay and the marriage bar.

## Women's work – carriage cleaning under nationalisation

The Transport Act of 1947 brought about the nationalisation of the railway network in Britain as part of Clement Attlee's nationalising of public services. The state-owned company British Railways was formed in 1948, operating overground rail transport across the country until it was privatised in stages between 1994 and 1997.^44^ Two important surveys of women's work took place during the period of nationalisation. There had been an increased interest in women's equality and employment issues during the 1970s and 1980s, a period which saw the growth of an active women's movement and pressure for equality for women. The focus of the first of these, the *Women and Employment Survey* (WES) of 1984 was on the place of employment in women's lives and questioned how far the situation of women was different from that of men. It found that a large proportion of women's occupations fell into a small number of categories – 30 per cent of these were clerical and 9 per cent unskilled.^45^ They summarised that the “majority of women were in secondary sector jobs with poor pay and no pensions, few opportunities for training or promotion and often little trade union representation”.^46^ In 1986, the Equal Opportunities Commission published its report of an investigation into equal opportunities for women in BR. The findings were similar: women accounted for only 6 per cent of the total number of BR staff; 61 per cent were in clerical positions; 25 per cent were in the low non-specialist conciliation grades; and less than 1 per cent were in operational roles such as footplate, guards, and signalmen.^47^

The second survey looked specifically at BR. Diana Robbins’ 1986 report provides a detailed investigation into equal opportunities for women in BR and is rich in both quantitative and qualitative data*.*^48^ One of the purposes of the Equal Opportunities report was to persuade managers and trade unions to “look at the evidence of employment disadvantage and discrimination facing women on the Railway, and to start the difficult process of change”. Its title is *Wanted: Railman*. The author had been astonished to see a blackboard at her local station with those two very words, “Wanted: Railman”. As she writes, “This was a recruitment advertisement by an equal opportunity employer for a job open to both sexes”.^49^ Where did this use of the masculine word “railman” come from? The employees of BR are divided into grade groups, each group having grade hierarchies built within it. The non-specialist occupations such as carriage cleaning can be found in the “general conciliation grades”, which are designated as “railman”, “leading or senior railman”, and “chargeman”. Robbins found in her research that most unskilled women manual workers had entered the general conciliation group and many remained there through their working life, as she points out a large proportion of these were carriage cleaners.^50^ The general lack of gender-specific data on the history of railway workers makes it difficult to identify problems of discrimination and therefore to argue a case for its elimination. However, Robbins found that in 1984 of the 1661 female Railmen, 77 per cent were carriage cleaners.^51^

In 1977, an agreement had been made between the BR Board and the unions that, in recognition of the Sex Discrimination Act of 1975, “all National, Regional, Divisional and Local Agreements referring to the male gender would equally apply to both men and women”. From then on BR referred to itself as an equal opportunities employer, but the above declaration was as far as it went. Although a proposal had been made in 1978 to adopt a “positive policy of equal opportunities’ and a possible programme for implementation”, the proposal was not pursued and by 1986 nothing further had been done to draw up a detailed policy or to oversee it.^52^

The Equal Pay Act, fully implemented from 1975, stipulated that men and women doing the same work at the same place of work should be paid at the same rate. Legislation could only be applied if that was the case. However, some jobs are subject to gender segregation where they are overwhelmingly done by men, while jobs in other occupations are overwhelmingly done by women. When the WES survey looked at occupational segregation it found that 63 per cent of women said they worked in jobs which were only done by other women, an important factor in gender inequality in the labour market with consequent implications for the gender pay gap.^53^*Wanted: Railman* also found a “considerable degree of job segregation by sex in BR” in 1981 and pinpointed two areas in particular, clerical work, and the women adult employees to be found in the general conciliation grades which included cleaners. There the group accounted for 25 per cent of women but only 15 per cent of men.^54^

As part of the survey, Robbins interviewed several carriage cleaners. The nature of the work appeared to have changed little since World War II. Although Harris (1997) writes that mechanised washing plants were generally introduced in the 1930s,^55^ this does not appear to be the experience of the carriage cleaners interviewed 50 years later. The work was largely manual with much use of buckets and ladders.

You have to climb up with heavy buckets of water. And if you are sweeping, you have to climb up and down all the time. You’ve got a trolley to help you on but not off. A *lot* of the girls have fallen between the trolley and the train and hurt their legs.^56^

There was little supervision or training and the equipment, although varying from place to place, was often old and sometimes inadequate. Although signalmen, for example, were entitled to overcoats, the carriage cleaners working in large, often cold, sheds were not. The work could also be dangerous when having to deal with hazardous cleaning chemicals. As noted, as part of their work, carriage cleaners may have come into contact with the residual diluted acid, the cleaning chemical Exmover. As one worker reported, “We have to climb all over the acid. The blockers won’t have it on their roads. And the drivers won’t touch it. *We* have to, yes”.^57^ The work too was often disagreeable. Wojtczak writes of having to deal with “all manner of disgusting items” including half-eaten food, vomit, and disposable nappies.^58^ The job can be so unpleasant that Robbins found that at one depot half the men cleaning the carriages were there for disciplinary reasons, the work being used as a punishment by putting men into a degrading and unpleasant situation.^59^ This is a useful illustration of the way in which “dirty work” is, as Simpson and Simpson put it, has “implications for prestige, work hierarchies, and social positioning”.^60^ On top of the disagreeable nature of the work, the report found that “women as a group are at a disadvantage” listing “indications of prejudice, of harassment, of unfair treatment, of … acts of injustice and potentially unlawful sex discrimination”.^61^

So why did the women put up with these poor conditions? Robbins and Wojtczak^62^ both mention that the shift work suited them; the hours were an attractive feature as they could be fitted around childcare commitments. Robbins adds that “Others – particularly black women with children – found that working permanently on nights at the sheds fitted in best with their other commitments”. The cleaners enjoyed the company of their fellow workers and found a great deal of camaraderie. Hilda Glassey worked alongside seven other girls and was “very happy”, she “enjoyed the work very much”.^63^ Laura Scott talks fondly of her lifelong friend Nan who she met when she was working as a carriage cleaner during World War II: “I wish I was working there now, I thoroughly enjoyed working for the railway with my friend Nan, we had a good laugh, I wish I was back, I am bored stiff.”^64^

Together the two reports of the 1980s, the WES and the Equal Opportunities Commission report, *Wanted*: *Railman,* present some insights into the work of female carriage cleaners. However, although the gender pay gap has fallen in the passing years, it has not ceased to exist and there is still a distinction today between “part-time” and “full-time” workers which acts to women's disadvantage. A contemporary report along the same lines as *Wanted*: *Railman*, but looking specifically at carriage cleaners, could help to valorise the work at the same time as offering a better understanding of pay and work conditions.

## Carriage cleaning under privatisation

The election of the Conservative Party under Margaret Thatcher in 1979 marked a major change in British politics. Not least is the idea that services should be run by the private sector and a deregulated economy should be left to the free market. Over the course of the 1980s, most nationalised industries and services were privatised, but the national rail network remained in public hands until the 1993 Railways Act was passed by the Government of John Major. In the run-up to privatisation John MacGregor (Secretary of State for Transport, 1992–94) envisaged that, in order to reduce costs, the rail industry would contract out to support services as much as practically possible.^65^ The NUR was strongly opposed to privatisation, partly in fear of the effects of franchising and there was a 24-hour strike against privatisation by the National Union of Rail, Maritime and Transport Workers (RMT) in April 1993. Later that month the dispute escalated and the RMT added a demand for a moratorium on BR contracting out business to the private sector.

Nevertheless, the Railways Act came into force in 1994. The next four years would see BR broken up into 100 individual companies. The responsibilities of each Train Operating Company (TOC) included rolling stock, stations and maintenance depots and the terms they took on gave them responsibility for light and heavy maintenance.^66^ As Charlton notes, privatisation saw many “ancillary service units detached from BR and sold to the private sector” and that much of the specialised expertise needed by the rail industry was now supplied by a “considerable battery of consultancy firms”.^67^ There were further disputes over pay and conditions and further strikes during 1995; rail privatisation was at the root of sporadic industrial unrest. The increase in the outsourcing of work resulted in carriage cleaners suffering from deterioration in employment terms and conditions.^68^ Because the majority of their employees were not railway workers they did not receive the benefits that had traditionally been enjoyed by their predecessors, free rail travel, the railway's pension scheme, and membership of an active union, for example.^69^ Fear about franchising had been realised.

Railway unions perform a crucial role in determining how far personnel policies can be altered to improve work conditions and opportunities for women, but few railway women had taken an active part in union activities during the twentieth century. Because carriage cleaners were concentrated in the conciliation grades, most women union members belonged to the NUR, but in 1978, women comprised only 5 per cent of NUR membership and it was not until 1982 that the first woman was elected to the National Executive Committee.^70^ In 1983, a motion had been discussed recommending the implementation of a policy to tackle “discrimination, disadvantage and harassment which women may encounter”. The motion was defeated. However, following the publication of *Wanted: Railman*, BR came under fire in the media and they acted swiftly. The Board was quick to appoint an Equal Opportunities Project team and to amplify its Equal Opportunities Policy Statement. *The Times* reported this with the headline “Male chauvinist BR vows to improve lot of its women workers”.^71^

After the NUR merged with the National Union of Seamen to form the RMT in 1990, union membership increased rapidly, particularly from the point when the prominent union leader Bob Crow was appointed as the General Secretary in 2002. Furthermore, the membership of women rose with many of these coming from Crow's championship of newer constituencies such as cleaning and catering.^72^ Crow was a strong negotiator and an enthusiastic advocate of cleaners, women, and black and ethnic minorities. His critics within the RMT felt that cleaners had “less strategic leverage” than those in some other occupations and that their employment turnover was greater than that of many RMT members. This could only preserve the power dynamics meaning that the cleaners, already the victims of an unequal hierarchical system, could be further penalised by their lowly position. Nevertheless, Crow made a strong defence of his strategy because of his strong belief in fighting low pay and poor conditions.^73^ It would appear that under the advocacy of Crow, the twenty-first century opened with an optimistic note in terms of pay and conditions for carriage cleaners and indeed Crow went on to fight for the rights of cleaners until his untimely death in 2014. However, in 2021, the RMT published a report, “Cleaning Up the Railways”, which found that cleaning was almost entirely outsourced and that nearly half the cleaners working on the network believed that there were not enough staff to do their jobs properly. The report concluded that “In spite of the essential and dangerous nature of their work, cleaners are still low paid and poorly treated, being paid on or around the National Minimum Wage and lacking sick pay”.^74^

Although a substantial amount of money is spent on cleaning trains, for many years, railway companies did not see any benefits in drawing attention to the work preferring instead to keep it hidden. However, the recent spread of the COVID-19 virus meant that it was in their interest to reassure passengers of the cleanliness of trains. As Leigh-Star argues, “infrastructure systems are often physically and metaphorically veiled beneath the surface of urban life. They only tend to become manifest when they cease to function or when the flows sustained by them are interrupted”.^1^ The COVID-19 pandemic meant that passenger numbers fell to levels never before seen. Passengers were fearful of their exposure to the virus on trains and were advised to stay at home and not to travel on public transport by the Government. During the peak of the lockdown, rail and underground usage was just 5 per cent of normal levels the crippling cost of this took its toll on TOCs. In an effort to encourage passengers to get back on board and to keep trains free from the virus, extra cleaning took place and was now very much in evidence with an increase in the number of board cleaners. When interviewed, cleaning specialist Shaun Doak of the firm React pointed on out that TOCs had taken the opportunity during lockdown to deep clean their trains and that practices had changed to include much more visible on-board cleaning. In his words “for far too long cleaning has almost been a forgotten art. It's very far down the pecking order, whereas now it's up top”. Part of the new cleaning regime was to take “swift action” after a passenger has a confirmed case of the virus. This involved decontamination of “not just the seat and carriage the passenger is believed to have been in but the entire train”. But, as Doak points out, “There are a lot of barrier products out there that make claims to protect against Covid-19 for any amount of days … My guidance to the TOCs is to do some due diligence on these products and analyse the relevant test data sheets”.^76^ Or to put it another way, there was no guarantee that the barrier products were effective and it could be argued, that cleaners were now being exposed to further danger at work, that of the COVID-19 virus.

This paper has revealed the consistently unpleasant nature of the work of carriage cleaners and some of the hazards it involves. It usually takes place at night and out of sight and partly because of this it has often been ignored, consequently, there is a limited understanding of the work and little acknowledgement of its importance by either railway companies or railway historians. Their work is riven with political issues affecting work conditions and wages. It is largely undertaken by working-class women and, more recently, migrant workers whose class, gender, and ethnicity give them little strategic leverage. Furthermore, the cleaners are often part-time workers disadvantaged by their part-time status and are stigmatized by the dirty nature of their employment. This only helps to further their invisibility. In many ways, little has changed since 1849 in terms of low pay, hard work, unpleasantness, danger, and invisibility, as such the work of carriage cleaners is a reflection of a hierarchical system which preserves the intersectional oppressions and inequalities of class, gender, and race. The railways constitute a system which is dependent on its cleaning labour force, but did not acknowledge its importance until it had to. For over 150 years cleaners were hidden from sight until it proved advantageous to bring them to the fore in order to maintain the financial viability of the TOCs.

This piece represents the first steps in overcoming the absence of information about carriage cleaners in railway history. An argument has been made for further research into carriage cleaning, and its role as part of the infrastructure of the railways from the nineteenth century onwards, offering a better understanding of pay and work conditions and helping to valorise the work. There is an absence of data relating specifically to women in this field; giving the opportunity to discover new historical sources and to create new material relating to the position of women as carriage cleaners today. Both would add a new dimension to current research. There is also much scope for further research in terms of the treatment of migrant railway cleaners in the twenty-first century and their support from the railway unions, as well as recent moves towards the use of robotics and autonomous systems in the railway cleaning sector.

## Notes

1 Susan Leigh-Star, “The Ethnography of Infrastructure”, *American Behavioural Scientist* 43:3 (1999), 377–91.

2 Leigh-Star, “Infrastructure”, 379.

3 Stephen Graham and Nigel Thrift, “Out of Order. Understanding Repair and Maintenance”, *Theory, Culture & Society* 24:3 (2007), 1–25, here 1–2.

4 Francis Head, *Stokers and Pokers* (London: John Murray Publishers, 1849).

5 Head, *Stokers and Pokers*, 8.

6 Verónica Caridad Rabeloa and Ramaswami Mahalingamb, “‘They Really Don’t Want to See Us’: How Cleaners Experience Invisible ‘Dirty’ Work”, *Journal of Vocational Behavior* 113 (2019), 103–114, here 104–5.

7 Ruth Simpson and Alexander Simpson, “‘Embodying’ Dirty Work: A Review of the Literature”, *Sociology Compass* 12:6 (2018), 1–9, here 2.

8 By the early twenty-first century the railway workforce had a high proportion of migrant workers, especially in London. Between 1956 and 1970, more than 4,000 staff had been recruited from the Caribbean to work on London's transport network as well as other employees from overseas, including Ireland and Poland.

9 Sheila Rowbotham, *Hidden from History* (London: Pluto Press Ltd, 1973).

10 Margaret Walsh, “Gendering Transport History: Retrospect and Prospect”, *Journal of Transport History* 23:1 (2002), 2–8, here 2.

11 Anne Munro, *Women, Work and Trade Unions* (Abingdon, Oxon: Routledge, 1999).

12 Caroline Criado-Perez, *Invisible Women: Exposing Data Bias in a World Designed for Men* (London: Vintage, 2020).

13 Helena Wojtczak, *Railwaywomen: Exploitation, Betrayal, and Triumph in the Workplace* (East Sussex: Hastings Press, 2005).

14 Philip Bagwell, *The Railwaymen: The History of the National Union of Railwaymen. The Beeching Era and After. Vol. 2* (London: George Allen & Unwin, 1982).

15 Head, *Stokers and Pokers*, 9.

16 Head, *Stokers and Pokers*, 50.

17 Hamilton Ellis, *Railway Carriages in the British Isles. From 1830 to 1914* (London: Allen and Unwin, 1965).

18 Frank McKenna, *Railway Workers* (London: Faber & Faber, 1976), here 272–73.

19 Leigh-Star, “Infrastructure”, 379–86.

20 Jane Humphries, “Women in Paid Work”, in June Purvis (ed.), *Women's History: Britain, 1850–1945 An Introduction* (London: Routledge, 1995), 85–105, here 100.

21 Ellen Jordan, *The Women's Movement and Women's Employment in Nineteenth Century Britain* (London: Routledge, 1999), 62–3.

22 Wojtczak, *Railwaywomen*, 52.

23 Wojtczak, *Railwaywomen*, 11.

24 “Women carriage cleaners. G. W. R. Co.'s new departure”, *Daily Citizen (Manchester)* 30 June 1913.

25 Wojtczak, *Railwaywomen*, 47.

26 Wojtczak, *Railwaywomen*, 52–53.

27 “Inquests”, *Worcester Journal* 18 August 1855.

28 “Carriage Cleaner killed”, *Derry Journal* 30 August 1905.

29 “Railway Carriage Cleaner Gets Compensation”, *Edinburgh Evening News* 24 June 1903.

30 Wojtczak, *Railwaywomen*, 139.

31 Rosa Matheson, “Women and the Great Western Railway With Special Reference to Swindon Works”, Unpublished PhD dissertation, University of the West of England (2002), here 239.

32 Matheson, “Women and the Great Western Railway”, 181.

33 Wojtczak, *Railwaywomen*, 172.

34 Susan Major, *Female Railway Workers in World War II* (Barnsley: Pen and Sword, 2018).

35 In the early hours of 11 May 1941, two 1,000 pounds bombs fell on one of the station platforms at King's Cross, damaging a train, destroying the booking hall, general offices and a bar, and killing 12 people.

36 National Archive of Railway Oral History, National Railway Museum, York, NAROH2001 – 174.

37 National Archive of Railway Oral History, National Railway Museum, York, NAROH2002 – 85.

38 *London, Midland & Scottish Railway Gazette* 12:126 (1923).

39 Colin Divall, “Civilising Velocity Masculinity and the Marketing of Britain's Passenger Trains, 1921–39”, *Journal of Transport History* 32:2 (2011), 164–91, here 170.

40 National Archive of Railway Oral History, National Railway Museum, York, NAROH2000 – 32.

41 Major, *Female Railway Workers*, 18.

42 National Archive of Railway Oral History, National Railway Museum, York, NAROH2003 – 24.

43 Major, *Female Railway Workers*, 29.

44 British Railways began trading as BR in 1965.

45 Jean Martin and Ceridwen Roberts, “Putting Women on the Research Agenda: The 1980 Women and Employment Survey”, *Twenty-First Century Society* 1:2 (2006), 129–148, here 138.

46 Martin and Roberts, “Putting Women on the Research Agenda”, 140.

47 Diana Robbins. *Wanted: Railman: Report of an Investigation into Equal Opportunities for Women in British Rail* (London: Equal Opportunities Commission, HMSO, 1986).

48 Robbins, *Wanted: Railman*, 81.

49 Robbins, *Wanted: Railman*, 1.

50 Robbins, *Wanted: Railman*, xv.

51 Robbins, *Wanted: Railman*, 71.

52 Robbins, *Wanted: Railman*, 2.

53 Martin and Roberts, “Putting Women on the Research Agenda”, 137.

54 Robbins, *Wanted: Railman*, xii.

55 Michael Harris, “Cleaners, Engine and Train”, in Jack Simmons and Gordon Biddle (eds), *The Oxford Companion to British Railway History* (Oxford: Oxford University Press, 1997).

56 Robbins, *Wanted: Railman*, 72.

57 Ibid.

58 Wojtczak, *Railwaywomen*, 280–281.

59 Robbins, *Wanted: Railman*, 71.

60 Simpson and Simpson, “‘Embodying’ Dirty Work”, 2.

61 Robbins, *Wanted: Railman*, 86.

62 Wojtczak, *Railwaywomen*, 281.

63 National Archive of Railway Oral History, National Railway Museum, York, NAROH2002 – 85.

64 Ibid.

65 David Parker, *The Official History of Privatisation: Vol. 2, Popular Capitalism, 1987–1997* (Abingdon: Routledge, 2012), here 461.

66 Clive Charlton, “The Structure of the New Railway”, in Roger Freeman and Jon Shaw (eds), *All Change: British Railway Privatisation* (New York NY: McGraw-Hill, 2000), 31–56, here 37.

67 Charlton “The Structure of the New Railway”, 52.

68 Tim Strangleman, *Work Identity at the End of the Line? Privatisation and Culture Change in the UK Rail Industry* (Basingstoke: Palgrave Macmillan, 2004), 139.

69 Wojtczak, *Railwaywomen*, 303.

70 Robbins, *Wanted: Railman*, 43.

71 “Male Chauvinist BR Vows to Improve Lot of its Women Worker”, *Times* 25 June 1986.

72 Female membership of the RMT rose from 6 per cent in 1994 to 11 per cent in 2010.

73 Gregor Gall, *Bob Crow: Socialist, Leader, Fighter: A Political Biography* (Manchester: Manchester University Press, 2017).

74 “Cleaning up the Railways. Why a Safe Railway Must Mean the End of Outsourced Cleaning”, *RMT Newsletter* 26 June 2021, https://www.rmt.org.uk/news/cleaning-up-the-railways (accessed 14 January 2023).

75 Leigh-Star, “Infrastructure”, 386.

76 Andrew Tunnicliffe, “Getting Back on Track After Lockdown”, November 2020 https://rail.nridigital.com/future_rail_nov20/track_and_trace_train_covid-19 (accessed 14 January 2023).
